# Could negative behaviors by patients with dementia be positive communication? Seeking ways to understand and interpret their nonverbal communication

**DOI:** 10.1111/nuf.12674

**Published:** 2021-11-23

**Authors:** Huey‐Ming Tzeng, Glenn Knight

**Affiliations:** ^1^ School of Nursing The University of Texas Medical Branch at Galveston Galveston Texas USA; ^2^ Training2Care (UK) Ltd Colchester UK

**Keywords:** Alzheimer disease, caregiver education, dementia, in‐service training, nonverbal pain communication

## Abstract

In interactions with caregivers, patients with dementia have communication challenges that are common and worrisome to families. Family and professional caregivers find it challenging to “guess” or “interpret” what their patients with dementia are trying to tell them. In this creative controversy article, we discuss how family and professional caregivers can seek to understand and correctly interpret the nonverbal communications of patients with dementia (behaviors, actions, facial expressions, and vocal sounds). Equipping family and professional caregivers with the resources to interpret the nonverbal communications of patients with dementia requires a commitment to in‐service and family education in healthcare facilities. Nurses could play a critical role in raising the awareness among the public about the potential changes and declines in verbal communications of the patients with dementia.

## 1 INTRODUCTION

This section of the Nursing Forum is set aside to allow authors to propose “wild ideas” for our consideration. A Creative Controversy aims to break with traditional thinking and pose a new way of considering an issue. Sometimes these ideas are a slight stretch of the imagination; other times, they are radical departures from the norm. Both are designed to stimulate conversation about a topic that concerns the profession. We are pleased to bring readers this Creative Controversy.

### 1.1 Problem

In interactions with healthcare providers, patients with dementia (Alzheimer's disease and others) have communication challenges that are common and worrisome to family caregivers.^1^ The challenges increase as dementia progresses, primarily because of the cognitive decline that affects a patient's ability to understand and verbally communicate their opinions and feelings (e.g., pain and discomfort), especially during end‐of‐life care.^2^, ^3^ However, clinical practice research into assessing the decision‐making capacity of dementia patients for healthcare and matters of daily living are limited.^1^

The World Health Organization^4^ urged the engagement of patients and their family members to support each patient's journey to safer healthcare and to ensure a constant flow of information between patients, family caregivers, and healthcare providers.^4^ Understanding the needs of patients with dementia who are nonverbal could mitigate the risk of such patients developing or being labeled as urine or feces incontinent.^5^ Edwards et al.^5^ called for action to incorporate verbal and nonverbal communication skills training for caregivers of patients with dementia.^5^

In this short article, we explore how family and professional caregivers can seek to understand and correctly interpret the nonverbal communications of patients with dementia (behaviors, actions, facial expressions, and vocalizations). One of the authors, a training expert for healthcare services, believes that learning how to interpret the nonverbal clues presented by patients with dementia, especially those in the late dementia stage, is crucial. Family and professional caregivers have to interpret those clues, often perceived as negative behaviors. Nonverbal behaviors in patients with dementia are often a way of delivering a message.^6^ In other words, patients with dementia, especially those in the late dementia stage, often communicate via nonverbal cues, and family and professional caregivers must be trained to interpret these cues. Actions are desperately needed to incorporate verbal and nonverbal communication skills training for family and professional caregivers of patients with dementia.^5^ However, based on the authors' personal experience and observations, family and professional caregivers of patients in the late dementia stage often overlook their patients' nonverbal cues. In some instances, family and professional caregivers find it challenging to “guess” or “interpret” what their patients are trying to tell them.

## 2 CHARACTERISTICS OF PATIENTS WITH DEMENTIA

### 2.1 Alzheimer disease stages

The Alzheimer's Association in the United States^7^ points out the changes in verbal communication that affect patients with dementia (Alzheimer and others)—specifically, increasing reliance on gestures rather than speech, and using speech less often. In the early Alzheimer stage, patients are still able to engage in social activities and conversation. In the middle stage, they can gradually demonstrate behavioral changes (e.g., repetitive hand‐wringing or tissue‐shredding). Difficulty in oral communication and self‐care increases. Giving patients visual cues and using written notes could help. Late‐stage Alzheimer's disease can last from several weeks to several years and requires 24‐h care, 7 days a week. Patients in the late stage might rely solely on nonverbal communication such as facial expressions and gestures.^7^

### 2.2 Walking in the shoes of people with dementia

In 2002, a national nonprofit organization with a mission to change the general public's perception of aging and dementia developed an in‐person simulation program called the Virtual Dementia Tour (Second Wind Dreams).^8^, ^9^ The Virtual Dementia Tour heightens awareness on the part of the family and professional caregivers about the communication and comprehension challenges experienced by dementia patients.^8^, ^9^ Caregivers can experience the degenerative physical symptoms common to patients with dementia (e.g., impaired vision and motor skills).^8^ Author 1 took the Virtual Dementia Tour in 2016 and, during the simulation, failed to accomplish any of the assigned tasks. It was indeed an eye‐opening experience to be in the shoes of someone with dementia!

#### 2.2.1 Can patients with dementia who are nonverbal communicate opinions and feelings?

In Reducing the Impact of Dementia in America: A Decadal Survey of the Behavioral and Social Sciences (p. 103),^1^ the daughter of an 82‐year‐old father with late‐stage Alzheimer disease tells of serious quality and safety concerns related to care transitions and the communication of pain during her father's cognitive decline.

Dad was returned to the memory care floor after a hospitalization. While we arranged ongoing 24‐h care, during an outing with a lay caregiver, this caregiver was concerned that Dad would fall while using the toilet, so he engaged with Dad physically. Dad fell against the bathroom sink and cracked two ribs. Dad was not able to verbally express his pain and he was not diagnosed right away. Ultimately Dad returned to the hospital, where he experienced delirium and received antipsychotic medication (abstracted for brevity).^1^

Patients with advanced dementia might still be able to say words or phrases but might experience increased difficulty in verbally communicating pain.^10^, ^11^ Caregivers could encourage nonverbal communication (e.g., asking patients to point and use touch, sights, or sounds).^11^ Still, are any resources available to help caregivers interpret body language, facial expressions, or negative vocalizations by such patients? Yes, although the interpretations are still developing.

### 2.3 Understanding nonverbal messages

The story of the 82‐year‐old father already mentioned^1^ underlines the frustration felt by family and professional caregivers and their need to interpret nonverbal communications (e.g., pain or discomfort expressed in behaviors and gestures) by patients with dementia. Ortega and Shin^12^ emphasized the importance of preparing clinicians to identify patients with non‐English‐language needs. It is critical to build a positive patient–clinician relationship in the setting of language discordance when the patient and the healthcare professional lack proficiency in the same languages. Also, clinicians need to navigate language assistance services and to communicate independently with the non‐English‐speaking people to whom they are providing direct care.^12^ However, communication with patients who have dementia and who no longer can communicate verbally is not mentioned.^12^ We recognize the need to communicate with non‐English speakers effectively. We need the same, if not more, initiatives to communicate with nonverbal patients with dementia.

#### 2.3.1 Can patients with dementia who are nonverbal communicate pain or feeling unwell?

For timely pain management, caregivers are in desperate need of a way to interpret the nonverbal communications of patients with dementia.^13^ Pain is highly prevalent in patients with dementia who are nonverbal. Chronic pain is still undertreated in such patients because of changes in their perception and expression of pain.^14^ Near the end of life, patients with dementia might have increased pain when resting.^13^ Pain severity could predict a decline in daily living functioning for those patients.^15^ A systematic review showed strong scientific evidence for five body movements as pain indicators in older adults who had cognitive impairments and who could not self‐report pain: physical aggression, agitation (restlessness), guarding, rubbing, and rigidity.^16^ However, additional studies are needed to validate the associations between pain in patients with dementia and their probably pain‐related body movements and behaviors.^16^

In any care setting, family and professional caregivers can use validated observational tools to help identify probable pain in patients with dementia who have limited ability to self‐report pain.^13^, ^17^, ^18^, ^19^ For example, the Pain Assessment in Advanced Dementia scale^17^ is a five‐behavior‐item observational tool with a total score ranging from 0 to 10 (0 = no pain, 10 = presenting pain behavior). Caregivers score what they see and hear with respect to breathing patterns, negative vocalization, facial expression, body language, and consolability for up to 5 min during and after active movement.^17^, ^20^ The scale was shown to detect significant differences in the scores before and after administration of pain medications.^17^ In another example, the Pain Assessment in Impaired Cognition^13^ instrument measures 15 items in three dimensions, for a total score ranging from 0 to 45 (0 = no pain, ≥3 = probably having pain).^13^ The three dimensions are facial expression (e.g., frowning, described as lowering and drawing brows together, narrowing eyes, raising upper lip, opening mouth, and looking tense), body movements (e.g., freezing), and vocalization (e.g., shouting).^13^

At home, family caregivers could potentially use validated observational tools (i.e., pain assessment in advanced dementia scale [PAINAD]; the revised pain assessment checklist for seniors with limited ability to communicate [PACSLAC‐II]) to facilitate earlier pain detection in community‐dwelling older adults with severe dementia.^19^ Evaluation of reliability showed that there were no statistically significant reliability coefficient differences between laypeople and caregiver staff working in the long‐term care facilities to the PAINAD and the PACSLAC‐II scores.^19^ In addition, healthcare facilities could adopt facial recognition technology to detect facial microexpressions related to pain while observers record the presence of other signs (e.g., whimpering and groaning; change in body language; and physical, physiologic, and behavioral changes).^21^, ^22^ The electronic Pain Assessment Tool^21^, ^22^ has good reliability to assess pain in patients with moderate‐to‐severe dementia. The electronic Pain Assessment Tool uses a 10‐s video, mapping the face of a patient with dementia to automatically identify the presence of pain in real‐time.

### 2.4 Internet resources for interpreting nonverbal communication

Family and professional caregivers can learn the “language” of patients with dementia.^8^, ^9^ The Dementia Dictionary^23^ website based in the United Kingdom continues to compile and suggest interpretations for the nonverbal communications of patients with dementia (behaviors, actions, noises, emotions, or situations, as witnessed by professional or lay caregivers). Each interpretation is developed by the Interpreter Forum and finalized by an advisory group of five to seven experienced dementia interpreters with dementia caregiving knowledge. The Dementia Dictionary^23^ website is made freely available, without login or registration requirements.

**Figure 1:**
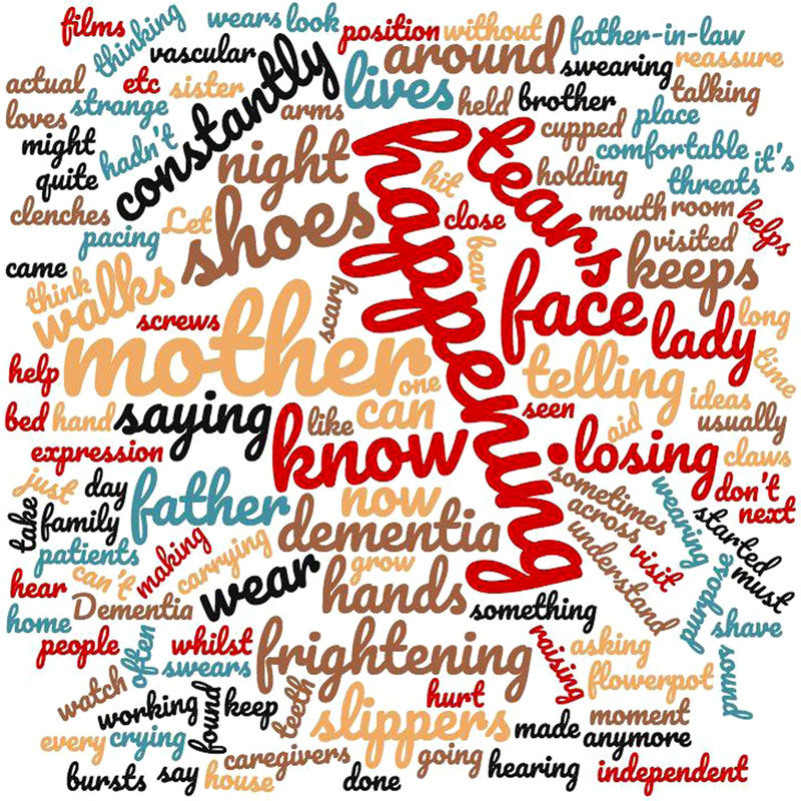
Word cloud using the words in the Dementia Dictionary's 13 request narratives related to pain [Color figure can be viewed at wileyonlinelibrary.com]

**Figure 2:**
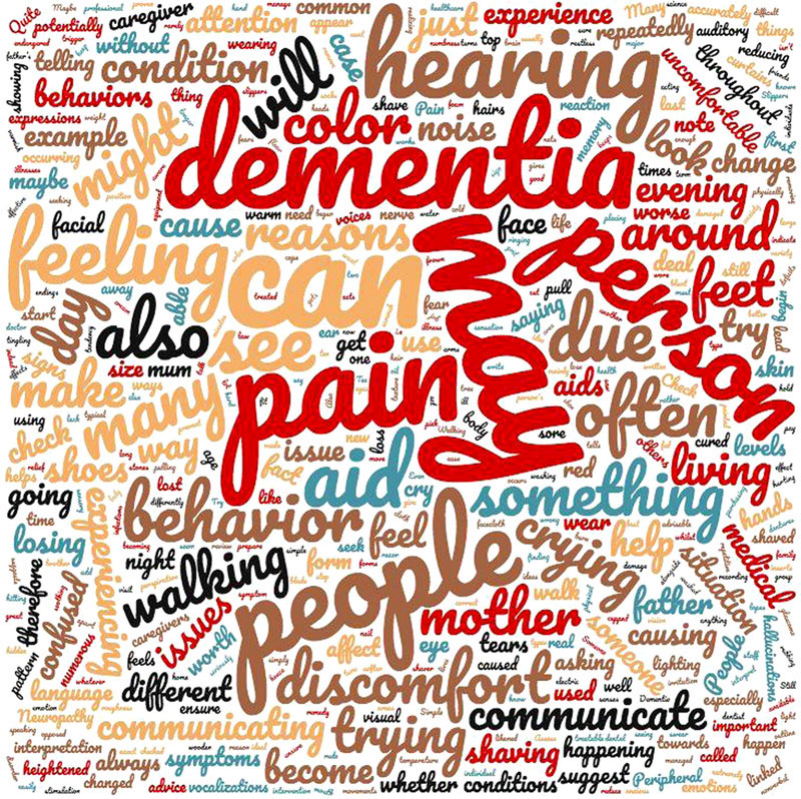
Word cloud using the words in the Dementia Dictionary's interpretation statements for the 13 request narratives related to pain [Color figure can be viewed at wileyonlinelibrary.com]

We used the keyword “pain” to search interpretations within the Dementia Dictionary,^23^, ^24^ locating 13 unique interpretation requests and corresponding interpretations that mentioned “pain” or “painful” feelings (Table S1). Of the interpretation requests, two had two interpretation statements each. Most of the posted interpretations have been written by professional caregivers. We prepared a word cloud (Figure 1) using the words in the 13 inquires (i.e., request narratives related to pain) for interpretation. A few words stand out: night, shoes and slippers, hands, face, tears, walking, and fright (felt by the caregivers). A second word cloud (Figure 2) uses the words in the interpretation statements. Several words stand out: pain, discomfort feelings, crying, walking and feet, and hearing. The words “pain” and “discomfort” seem to be used interchangeably in the interpretation statements. The interpretations of nonverbal communications by patients with dementia using Internet resources such as the Dementia Dictionary^23^, ^24^ are based on intentional observation.

During a professional meeting with one of the authors in 2021, a dementia educator and trainer for healthcare services, who lives in the southern United States, stressed that it is challenging for healthcare professionals to recognize whether patients with dementia who are nonverbal are experiencing pain or discomfort. It is even more challenging for hospital staff, given a short length of stay. But even when discomfort is acknowledged in a patient with dementia, it is often overlooked or ignored because it is only “discomfort,” and not “pain.” This dementia educator emphasized how preconceptions might influence the individual interpretations of such nonverbal communication.

## 3 CONCLUSIONS

Communication skills training for family and professional caregivers of patients with dementia, especially patients who have difficulty with verbal communication, has to be developed in partnership with family caregivers and healthcare staff.^5^ For example, the pain felt by patients with dementia who are nonverbal is real and is associated with symptoms of psychosis (for instance, delusion).^25^ Before a decision is made to treat symptoms of psychosis either pharmacologically or nonpharmacologically, regular pain assessments and timely recognition of expressions of pain in patients with dementia (e.g., change in nonverbal communication, and agitated behavior) should be the rule and part of the clinical practice.^25^, ^26^

During nursing home stays, patients with dementia might exhibit unusual symptoms (e.g., pain and agitation) and receive less‐than‐optimal symptom control from admission to end of life.^27^ Equipping healthcare professionals with the resources to understand the nonverbal communications of patients with dementia (such as “pain recognition”) accords with a commitment by healthcare facilities for in‐service education.^12^, ^21^, ^22^, ^28^ In addition, family caregivers should be supported with access to resources such as the Alzheimer's Association^7^ and the Dementia Dictionary^23^ websites that will help them understand and interpret the nonverbal communications of their loved ones with dementia.^29^ Also, we need to raise awareness among the public about the potential changes and declines in verbal communications of patients with dementia.^7^ Nurses could play a critical role in raising awareness in the medical and healthcare settings across the care continuum, including outpatient and community settings (e.g., lifelong learning institutes for retirees and community centers). Patients with dementia still can feel and sense! They could still have a strong desire to connect and communicate with their loved ones and professional caregivers. Are we here to listen to the nonverbal communications from patients with dementia? Yes, we are! We determine to hear the nonverbal communications of our patients or loved ones with dementia who may have lost their voices partially or entirely.

## CONFLICT OF INTERESTS

The authors declare that there are no conflict of interests. Author Glenn Knight is the Chief Executive Officer of Training2Care, a care‐specific training provider in England, delivering more than 160 different courses. Training2Care is the UK partner of Second Wind Dreams, which delivers the inspirational Virtual Dementia Tour.

## Supporting information

Supplementary Information
