# Supplementary material for: Could negative behaviors by patients with dementia be positive communication? Seeking ways to understand and interpret their nonverbal communication
Source: Nurs Forum. 2021 Nov 23;57(2):318–22. doi: 10.1111/nuf.12674 (PMC9299486; doi:10.1111/nuf.12674)
Supplement: Supplementary file 1 — Supplementary Information [file NUF-57-318-s001.docx]

Supplemental TABLE 1 The 13 request narratives related to pain and their interpretations in the Dementia Dictionary24,a

| Request title | Request narrative | Interpretation |
| --- | --- | --- |
| 1. My mother keeps misplacing her hearing aid | My mother wears a hearing aid, and she keeps losing it. When it is found, it is often in a strange place such as a flowerpot. I am now thinking she might be losing it on purpose. | She is telling you that the hearing aid is painful to wear. Is your mum’s hearing aid that loops at the top of the ear? It may be rubbing! Your mum losing or misplacing the hearing aid may be due to discomfort, although my mum just turns hers off as she is fed up with all the noise around her! Is the hearing aid comfortable for your mum to wear? As we age, our ear shape can change. Might it be worth getting a refit done? Also, check for sore areas. I wear glasses and have issues sometimes with the area at the top. Please always check with qualified medical staff such as an audiologist if you feel that a hearing aid is causing discomfort.  (Posted on February 12, 2021. This interpretation was provided by a lay caregiver (R. Gates) and has been modified and paraphrased as needed to fit into the table format.)  The color of a hearing aid and size are major issues that need to be addressed for people with dementia. Hearing aids always seem to be skin-colored, which is not great for people with eye conditions such as glaucoma or cataracts. Many people with dementia will experience these types of eye conditions and, therefore, will not be able to see the color or outline of the hearing aid due to the color and size. Some people and families prefer hidden hearing aids. These are just not ideal for people who have difficulty seeing due to eye conditions. We always recommend a larger hearing aid as people’s journey of dementia continues. The color is the next problem, and from the age of around 40, most people’s eyesight will be impaired, and color vision starts to deteriorate. The first color we learn in life is red, and the last color we lose is red, so we must look at making hearing aids in different colors to stand out and make the difference. I experienced this with a relative that wore a hearing aid that she kept losing, and I saw that she couldn’t see it due to blending in on magnolia or wooden surfaces. I therefore painted one red with nail varnish, and she rarely misplaced this. Your mother’s behavior is telling you that she cannot see the hearing aid due to color and size. Things you may try including speaking to your mother’s audiologist about colored hearing aids as these are now available in the marketplace and ensure that the hearing aid is large enough to be able to see easily.  (Posted on February 19, 2021. This interpretation was provided by a professional caregiver (G. Knight) and has been modified and paraphrased as needed to fit into the table format.) |
| 2. Threats | My father-in-law has made some quite frightening threats, and I don’t know why or what he is telling me. Why is this happening? He has vascular dementia and lives on his own. | With dementia, behaviors often change. Understandably these can be hard for family and friends to deal with. Quite often, the frontal lobes are affected. In turn, this changes the personality. The frontal lobes also control our impulses. People who have deficits here may act aggressively, insensitively, and rudely. Your father-in-law could be trying to communicate that something feels wrong with him. These behaviors are due to real issues and not because he is deliberately trying to be difficult. The behavior your father-in-law is showing could be a way of him communicating that he may be in pain, confused, or frightened. Consider a support group, where you can learn about helpful strategies from others experiencing the same issues. It is also worth getting a medication review.  (Posted on February 13, 2021. This interpretation was provided by a professional caregiver (S. Stringer) and has been modified and paraphrased as needed to fit into the table format.) |
| 3. Physical aggression | Dementia patients hit caregivers. | This situation could be due to the approach of the caregiver toward the person with dementia. It could be that the person with dementia is reading this person’s nonverbal language (i.e., body language) and feeling threatened by the caregiver somehow. The caregiver could appear intimidating toward the person without realizing it. The person with dementia may fear losing privacy and dignity, depending on the situation. This could also be because the person with dementia feels they are losing control but cannot communicate their needs to the caregivers. The person with dementia could be hitting caregivers with frustration. Check to see if the person with dementia was in any pain too.  (Posted on May 27, 2021. This interpretation was provided by a professional caregiver (N. Roberts) and has been modified and paraphrased as needed to fit into the table format.)  If this is a new behavior, check whether the patients with dementia communicate they might be in pain. Pain often gets overlooked for people living with dementia, especially if they express their pain nonverbally.  (Posted on June 3, 2021. This interpretation was provided by a professional caregiver (M. Peachey) and has been modified and paraphrased as needed to fit into the table format.) |
| 4. My mother started swearing | My mother has started swearing, and I do not know why. I keep asking her, and she just swears at me. Why is she doing it, and what should I do? | Could your mother say that she is in pain or discomfort, or she is feeling unsettled or confused? It might not be immediately apparent what the issue is, but we often use swearing as an expression of emotion or a reaction to pain. It could be related to loss of inhibition. Your mother might not know whether it is appropriate to swear or how it might affect you. Your mother may be expressing: “I am feeling confused and anxious.”  (Posted on February 17, 2021. This interpretation was provided by a professional caregiver (M. Rowland) and has been modified and paraphrased as needed to fit into the table format.) |
| 5. Lady crying in the evenings | I am working with a lady with dementia at the moment. She is very independent, helps in and out of the home, loves to watch films, etc. But now and then, she bursts into tears. When I say tears, it’s more the sound of crying, no actual tears. | There are many reasons why people with dementia make a crying noise but without tears. Here I am going to look at sundowning as you have mentioned that this happens in the evenings. Please note, however, that crying with or without tears can happen at all hours of the day and sometimes continue throughout the day. The first thing to note is that people with dementia still have emotions and add this to short-term memory loss. They may start to cry and then “forget” why they are crying, hence no tears and just the noise. If this is generally happening in the evening, we need to investigate if this is caused by a common issue called sundowning. As the evening draws in or at dusk, they may start to experience heightened anxiety, possibly agitation, and fear. One of the reasons for this could be caused by tiredness. People with dementia have so many issues with sensory processing during the day that they feel exhausted and tired when evening comes. In the evening, the natural light reduces, reflections can appear in windows, shadows form, and they may have heightened reactions to noise levels. In a busy nursing home with buzzes, alarms, phones, TVs, radios, hoovering, and so on, this will be a very stressful time and potentially lead to fear. Still, today science cannot explain why people cry apart from an emotional response to the environment, situation, pain, or a multitude of emotions. However, it has been proven that in children, crying can be used as a form of attracting attention, and indeed throughout our lives, we know that we become the center of attention when we cry. In this case, I will suggest that the crying is linked to sundowning as it is occurring in the evening. This lady’s crying could tell you that she is fearful of something and is trying to keep you with her by crying to seek attention and reassurance. Things to do to help include closing curtains or using nets curtains to reduce reflections, reducing sound levels, ensuring that lighting is bright, and potentially using SAD (seasonal affective disorder) lighting.  (Posted on February 20, 2021. This interpretation was provided by a professional caregiver (G. Knight) and has been modified and paraphrased as needed to fit into the table format.) |
| 6. My grandmother keeps saying she can hear people talking in her house at nighttime | She keeps saying that when she is in bed at night, she can hear people in her house talking in the next room, which must be very frightening for her. What should I do to reassure her, and what is happening to her? | Changes in the brain with dementia can often cause hallucinations that affect the senses. These tend to affect the visual or auditory. Although there may be no additional reason for your grandmother saying she hears voices, these voices are very real to her. What we can do is to deal with them. First, check to make sure she isn’t in any pain, as this is known to cause auditory and visual hallucinations. Reassure her in a supportive way. Look for a pattern. Does this happen when she does a certain activity before bedtime, or has her routine changed? Try removing possible triggers; maybe background noise could be causing the problem.  (Posted on February 13, 2021. This interpretation was provided by a professional caregiver (S. Stringer) and has been modified and paraphrased as needed to fit into the table format.) |
| 7. My father doesn’t like being shaved | My father lives with me and can’t shave his face anymore. Do I take him to a barber to have it done? Let him grow his beard out? What other ideas? | There are numerous reasons why individuals may not like being shaved. It is important to pay attention to exactly what behaviors the person is showing when this person is being shaved to accurately identify the exact cause of their dislike. Many people may try to pull their heads away, scrunch their eyes up, frown, and use their hands to physically stop you from shaving. If this is the case, my interpretation would be that the person does not want to shave because they find it painful. A simple change that can be made to remedy this would be to ensure that the equipment used is in good shape and correct for the job. For example, a blunt blade will pull at hairs rather than cut them, and using an electric shaver on hairs that are too long will do the same thing. The person may also be experiencing pain because of a physical condition that they are living in. Peripheral neuropathy can cause numbness, tingling, and pain, and occurs when the nerve endings become damaged. It usually affects the feet, hands, and arms but can also affect the face. If someone is experiencing pain on their face, shaving can make this sensation feel ten times worse. Simple things like washing the face with warm water, wringing out the facecloth, then placing the warm cloth over their face for a few minutes can help prepare their skin for shaving. This extra step helps soften the facial hair and helps prevent pulling when you shave. Tea tree oil shaving cream can also be fantastic to help ease some of the uncomfortable sensations that people may feel, as it acts as an anti-inflammatory so has a soothing effect on the skin. It works especially well to deal with razor burn.  (Posted on February 14, 2021. This interpretation was provided by a professional caregiver (M. Peachey) and has been modified and paraphrased as needed to fit into the table format.) |
| 8. Hand holding face | A lady is constantly holding her face with one hand, usually across her mouth. | Quite often, people with dementia can become restless and may make repetitive movements. This can be for a variety of reasons, such as pain or discomfort. Or it can be due to lack of stimulation. This lady could be trying to communicate that they are in pain. The fact that the hand keeps going to the mouth could signify she is having dental pain. Check to see when her last visit to the dentist was. Has the person lost weight? Maybe her dentures are no longer fit comfortably.  (Posted on February 25, 2021. This interpretation was provided by a professional caregiver (S. Stringer) and has been modified and paraphrased as needed to fit into the table format.) |
| 9. Cupped hands | My mother walked around with hands in a cupped position and held close to her as if carrying something. | A lot of people hold their hands in a cupped position when they are asking for something. As your mother is doing this while walking around, the two of these behaviors may be linked. Someone living with dementia may walk around for many different reasons. These reasons could be feeling lost, being unsure of what to do, being confused about something, being in pain/discomfort, and many others. Your mother may be asking for help with something. To more accurately interpret this behavior, we need to get some ideas about what the person might be asking for. The best way to do this is by recording when and where the behavior is happening and seeing if there is some pattern. We can also look at other things, such as her body language, facial expressions, and any vocalizations your mother may make, which could give us a better idea of what she is communicating.  (Posted on April 19, 2021. This interpretation was provided by a professional caregiver (M. Peachey) and has been modified and paraphrased as needed to fit into the table format.) |
| 10. Raising hands and clenching of teeth | My mother sometimes screws her face up into a scary expression and clenches her teeth while raising her arms and making her hands look like claws. She did this when my sister and her family came to visit. She hadn’t seen them for a long time. | Your mother could be communicating that she is in pain. Assess for pain and have her checked out by her doctor if it continues.  (Posted on May 12, 2021. This interpretation was provided by a professional caregiver (P.K. Beville) and has been modified and paraphrased as needed to fit into the table format.) |
| 11. My brother walked around with no shoes on | I do not understand. When I have visited my brother, he is pacing up and down without any shoes on. I do not know why this is happening when he has slippers to wear. Why is this happening, and how can I help him to know to wear shoes. I think he is going to hurt himself. | This situation could be that your brother is trying to communicate that wearing shoes is very uncomfortable or maybe hurting his feet. By walking on the cold floor gives him some relief from the irritation he could be feeling. People living with dementia tend to remove their shoes and socks due to a condition called peripheral neuropathy. This behavior may signal nerve damage that can cause pain in the feet; it could feel like having stones or grit in the shoes. Slippers are an issue in themselves. Once washed a few times, they can become uncomfortable on the feet due to the roughness or texture.  (Posted on February 15, 2021. This interpretation was provided by a professional caregiver (T. Waller) and has been modified and paraphrased as needed to fit into the table format.) |
| 12. My aunt doesn’t like wearing shoes, only her slippers | Is she saying she is more comfortable wearing slippers than shoes? | Your aunt could be saying she is more comfortable wearing her slippers. This behavior could be because she is experiencing some pain or discomfort in her feet, and the shoes make this worse. In this case, my interpretation would be, “my feet are sore, and these shoes are making it feel worse.” It might be worth seeking medical advice to see if anything is going on with her feet. If it is a treatable condition, then a medical intervention could solve the issue. If it cannot be cured, we can look at other ways of managing it. For example, such a condition could be addressed simply by purchasing some memory foam shoe inserts, which can effectively reduce the painful/uncomfortable feeling.  (Posted on June 3, 2021. This interpretation was provided by a professional caregiver (M. Peachey) and has been modified and paraphrased as needed to fit into the table format.) |
| 13. My father constantly walks day and night | My father constantly walks day and night, up and down. Why is this happening, and what is this telling me? | There will be many reasons why someone living with dementia may begin to walk around repeatedly, and it is a very common behavior that we see in many different people. An important clue as to what the person is trying to communicate is what time of the day they are repeatedly walking about. For example, if people are repeatedly walking about at night, this could be a completely different communication than someone doing this during the day. In your father’s case, the repeated walking is occurring both during the day and at night as well. This behavior suggests that whatever is causing this is happening throughout the day and night. If this is a new behavior, then this may indicate that something has changed for this person, therefore acting as a trigger for this behavior. Your father may have begun to experience something that is causing them some form of pain or discomfort, and that they are walking around to try to manage this. |
|  |  | Pain, illnesses, and infections often go undiagnosed in people living with dementia. The delay in being diagnosed is mainly down to the fact that they may communicate this differently to how we would, and the forms of communication used often get written off as just another symptom of dementia. Walking repeatedly is a typical example of this, as many people will often just say that they are doing this because of their dementia. If we write off this behavior in this way, then we may not pick up that someone has an underlying illness or condition which, if not treated, could lead to the person’s health becoming seriously endangered. Even if the underlying condition cannot be cured, there may still be numerous ways of managing this for the individual. It is prevalent for people to move around when they begin to experience pain or discomfort. For many people, this can help them to cope with the feeling they are experiencing. It could also be that they walk to try and get away from the pain or discomfort for some people. I would suggest talking to your father to see whether he is feeling in pain or is unwell. We can also lookout for any other signs or symptoms that the person may have become ill or in pain, such as how they are walking, their temperature, perspiration levels, facial expressions, vocalizations, and many other signs and symptoms. If your father tells you he is in pain or is feeling unwell, or if you see any signs and symptoms alongside the walking that may suggest this, it is advisable to seek further medical advice from a healthcare professional.  (Posted on February 15, 2021. This interpretation was provided by a professional caregiver (M. Peachey) and has been modified and paraphrased as needed to fit into the table format.) |

a The owner of the Dementia Dictionary website23,24 granted permission to author 1 to use content from the website.
